# Comparative analysis of robotic assisted versus laparoscopic Roux-en-Y gastric bypass: a retrospective cohort study

**DOI:** 10.1007/s11701-026-03734-6

**Published:** 2026-07-30

**Authors:** Matthew L. Brengman, Prajakta H. Waghmare, I-Fan Shih

**Affiliations:** 1 Advanced Surgical Partners of Virginia, Richmond, VA 23294 USA; 2 Intuitive Surgical Inc., Sunnyvale, CA USA

**Keywords:** Metabolic and bariatric surgery, Gastric bypass, Minimally invasive surgical procedures, Robotic surgical procedures, Laparoscopy

## Abstract

Comparing perioperative outcomes of robotic-assisted (RAS) and laparoscopic (LAP) Roux-en-Y gastric bypass (RYGB). A retrospective cohort study was conducted using Premier Healthcare Database, from 2020 to 2023. The study included adult patients with Body Mass Index 30 or higher who underwent elective inpatient RYGB surgery via RAS or LAP. RAS and LAP cohorts were matched through propensity scoring. Regression models with standard errors clustered by matched pair were used to estimate associations between surgical approach and perioperative outcomes. After matching, RAS-RYGB was associated with lower odds of conversion to open surgery (OR, 0.48; 95% CI, 0.32-0.73), ICU admission (OR, 0.57; 95% CI, 0.45-0.73), blood transfusion (OR, 0.77; 95% CI, 0.63-0.95) compared with LAP-RYGB. RAS-RYGB was also associated with slightly lower odds of being in a higher length-of-stay category (OR, 0.85; 95% CI, 0.81-0.89) and have 30-day all-cause reencounters (OR, 0.87; 95% CI, 0.82-0.91). Operating room time was longer with RAS-RYGB (mean difference, 17.5 min; 95% CI, 16.1-18.9), and bowel obstruction was more frequent (OR, 1.25; 95% CI, 1.01-1.54). Other perioperative complications were similar between groups. RAS-RYGB was associated with lower conversion to open surgery, blood transfusion, ICU admissions, modest shift towards shorter length of stay and post-operative visits than LAP-RYGB, but with higher bowel obstruction and longer operating time.

**Supplementary Information:**

The online version contains supplementary material available at 10.1007/s11701-026-03734-6.

## Introduction

Obesity is a global epidemic with rising prevalence, contributing significantly to morbidity and mortality worldwide [1, 2]. It is a well-established risk factor for numerous health conditions, including diabetes mellitus, cardiovascular diseases, and certain types of cancer [3]. According to Centers for Disease Control and Prevention (CDC), 1 in 5 children and 2 in 5 adults have obesity with obesity accounting for approximately $173 billion in annual health care costs [4]. 

Metabolic and bariatric surgery (MBS) is a well-established treatment for obesity and overweight, when nonoperative approaches have failed. Roux-en-Y gastric bypass (RYGB), is widely recognized as an effective long-term treatment for severe obesity and its related comorbidities [5]. According to the American Society for Metabolic and Bariatric Surgery (ASMBS), annual RYGB volume in the US increased from 41,280 cases in 2020 to 63,132 cases in 2023 [6]. RYGB alters both satiety and baseline hunger by altering the size of the stomach and bypassing the duodenum and proximal jejunum. The resulting mechanical and hormonal changes help to decrease caloric intake, resulting in significant weight loss [7]. 

Minimally invasive surgery (MIS) by laparoscopy (LAP) is the standard approach to perform RYGB. LAP is associated with reduced post-operative pain, shorter hospital stays, and fewer wound complications as compared to open procedures [8, 9]. The robotic-assisted surgical (RAS) approach is being increasingly adopted [10]. Early studies show RAS to be safe and effective [11, 12]. One meta-analysis reported fewer anastomotic stricture events, a lower reoperation rate, and decreased length of stay (LOS) with RAS [13]. However, the data is not uniformly positive with a more recent meta-analysis of a randomized control trial (RCT) and single institution studies reporting that RAS had a higher reoperation rate with similar complications as LAP [14]. 

Our objective was to compare perioperative complications and hospital resource utilization in LAP and RAS-RYGB surgeries using a contemporary all-payer US hospital discharge database. This data source allows analysis of a large and diverse patient population while incorporating stapler reinforcement, surgeon volume, and hospital volume into propensity score matching to provide a more comprehensive assessment of perioperative RYGB outcomes.

## Methods

This study was a retrospective, comparative outcomes study utilizing data from the Premier Healthcare Database (PHD) [15]. 

### Data source

PHD is a large, US hospital-based, Health Insurance Portability and Accountability Act (HIPAA)-compliant database containing inpatient and outpatient data from diverse hospitals and healthcare systems. It includes more than 135 million inpatient admissions, representing 25% of annual US inpatient admissions. In the US, retrospective analyses of PHD data are considered exempt from formal consent and institutional review board (IRB) approval pursuant to 45 CFR 46.101(b)(4).

### Study population

**Fig. 1:**
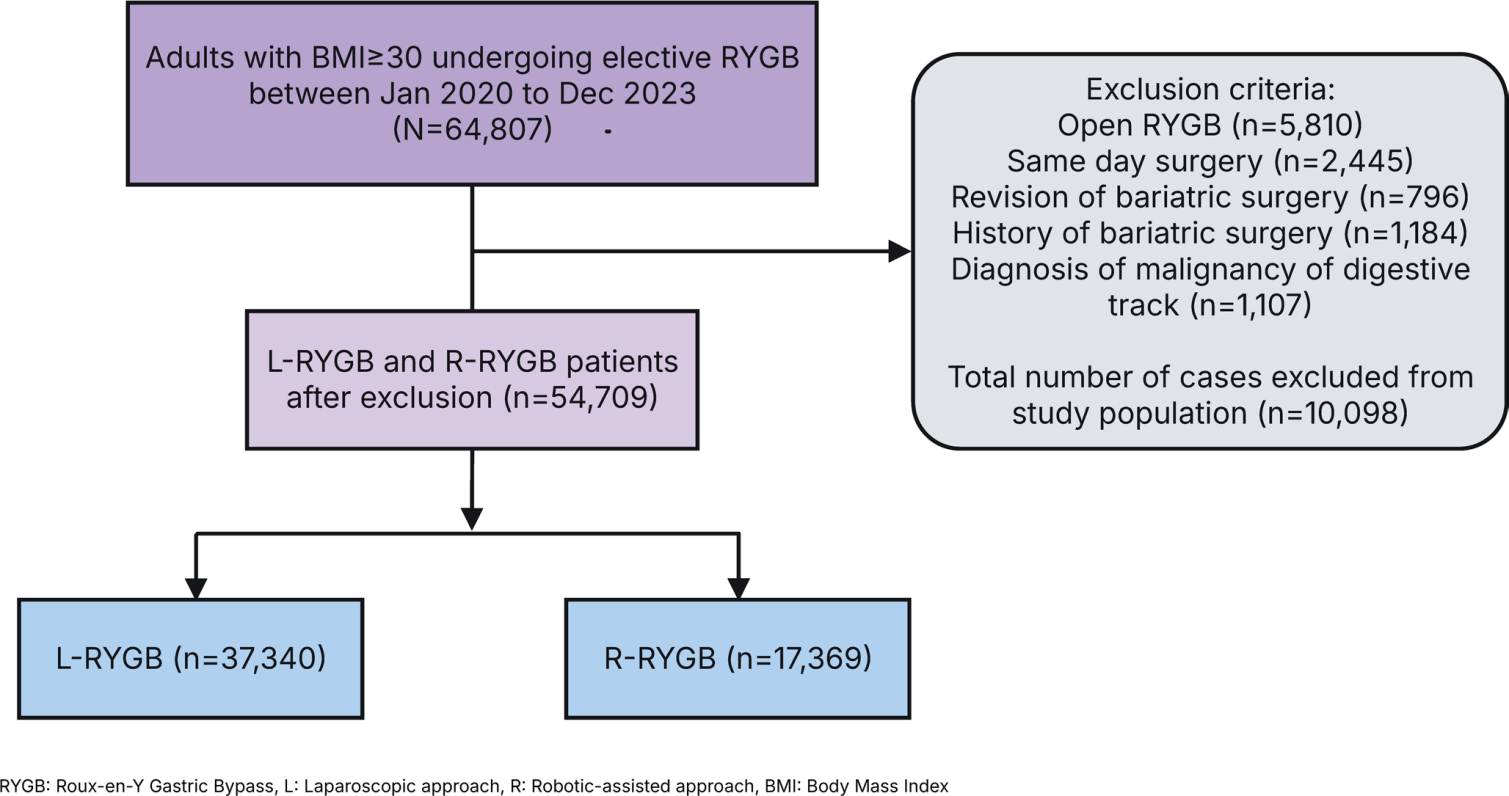
Study cohort selection flow diagram

Adult patients with a BMI of 30 or higher who underwent elective LAP or RAS-RYGB from January 2020 to December 2023 were included. The PHD database definition of elective admissions is based on the Centers for Medicare and Medicaid Services (CMS) Uniform Billing-04 admission type. Current Procedural Terminology (CPT) and International Classification of Diseases (ICD-10) Procedure Coding System (PCS) codes were used to identify LAP, RAS and open RYGB (Appendix A). Patients with open RYGB cases, same day discharge, revisional procedure, prior history of MBS and malignancy of digestive tract were excluded (Fig. 1, Appendix B). Missing patient demographic data was classified as unknown and included in the analysis.

### Patient and provider characteristics

Patient characteristics included age, gender, marital status, race, ethnicity, year of surgery, obesity status, Medicare Severity Diagnosis Related Groups (MS-DRGs) and Charlson Comorbidity Index (CCI) [16]. Other comorbidities commonly found in bariatric patients were included through CPT/ICD-10-Clinical Modification (CM) for diabetes, liver disease, gastroesophageal reflux disease (GERD), hypertension, hyperlipidemia, hernia diagnosis, sleep apnea, and the use of nicotine. Concomitant procedures of hernia repair including ventral or hiatal hernia repair, cholecystectomy, and lysis of adhesions diagnosis with procedure were included through CPT/ICD-10-PCS codes (Appendix C, Appendix D).

Hospital characteristics included primary insurance payer, hospital volume, region, number of beds, teaching status, urban/rural status. Hospital region was categorized using Census Regions and Divisions of the United States [17]. Hospital volumes were calculated annually as the total number of RYGB procedures for all surgical modalities within a given hospital. Surgeon characteristics included surgeon volume and surgeon specialty. Surgeon volume was calculated individually for each patient and estimated as the number of RYGB procedures performed by a given patient’s surgeon in the 12-month period prior to the date of surgery and were calculated separately for each modality (LAP and RAS). Hospital and surgeon volumes were stratified into three groups by low, medium and high volume tertile. Stapler and buttress use was identified through billing descriptions in the database and classified as stapler with buttress, stapler without buttress and no stapler use. They were included as matching covariates because prior bariatric surgery studies have reported associations between staple-line reinforcement and perioperative outcomes [18–20]. 

### Complications and outcome variables

We measured LOS, operating room (OR) time, conversion rates, and intensive care unit (ICU) admission during the surgery. Hospital LOS was calculated using the number of days from admission to discharge. Conversion rates from LAP/RAS to open were calculated through ICD codes (Appendix A). ICU admissions were identified through room and board billing for ICU after the day of surgery. Reencounters, reoperations and all-cause readmissions were measured. Reencounters were defined as the same facility healthcare encounters occurring within 30 days after the operation, including emergency department visits, urgent care visits, clinic encounters, observation stays, and inpatient readmissions. MBS-specific complications included anastomotic leak, bleeding, surgical site infection, sepsis, bowel obstruction, and blood transfusion and were defined using ICD and CPT codes. (Appendix E)

### Statistical analysis

The proportion of patients was analyzed based on surgical approach and years (2020–2023) by LAP and RAS modality. Propensity score matching (PSM) was performed to create study cohorts [21]. Patient characteristics, patient comorbidities, hospital, and surgeon characteristics, substance use, concomitant procedures and the type of stapler were included as covariates to perform propensity matching (Appendix C). Bivariate descriptive comparison was performed on various patient, hospital and surgeon characteristics using chi-square test at baseline before and after propensity matching. A nearest greedy matching 1:1 was performed without replacement, with a maximum allowable difference in propensity scores of 0.01 between matched subjects. Additionally, covariate balance was evaluated using *p-*value and standardized mean differences (SMD) before and after matching. Regression framework (logistic for binary outcomes, linear for continuous outcomes, proportional odds for LOS category) with standard errors clustered for matched-pair was applied. Odds ratios (OR) with 95% confidence intervals (CI) were reported. To address residual imbalance in surgeon volume and admission year after PSM matching, these two covariates were included in all outcome models.

Subgroup analysis was conducted on patients without concomitant hernia repairs and patients with class 3 obesity (BMI ≥ 40) using identical regression frameworks. Statistical significance was set at *p* < 0.05. All analyses were performed using R version 4.4.1. The manuscript followed the checklist from the Strengthening the Reporting of Observational studies in Epidemiology (STROBE) guidelines (Appendix F).

## Results

**Fig. 2:**
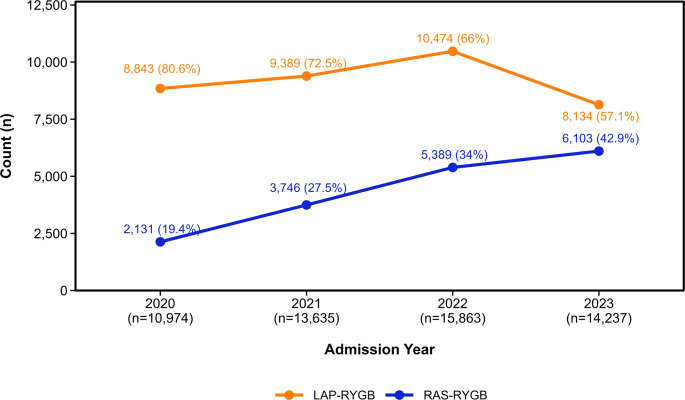
RYGB volume trends of LAP-RYGB and RAS-RYGB

After applying inclusion and exclusion criteria, the final study cohort comprised 54,709 patients with 37,340 LAP-RYGB and 17,369 RAS-RYGB (Fig. 1). Over the study period, there was an increasing volume and proportion of RAS-RYGB, with a corresponding decreasing proportion of LAP-RYGB (Fig. 2).

The baseline characteristics before propensity matching comparing RAS and LAP are noted in Table 1. Patients in the RAS cohort had a higher rate of concomitant hiatal hernia repair (22.0% vs. 19.5%, *p* < 0.001) and cholecystectomy (2.9% vs. 2.5%, *p* = 0.013) than LAP. Additionally, higher proportion of RAS-RYGB were done without staplers (27.7% vs. 23.0%) and staplers without reinforcement line compared to LAP-RYGB (65.5% vs. 59.3%, *p* < 0.001).

**Table 1:** Patient, Surgeon and Hospital-Related Characteristics in Roux-en-Y gastric bypass patients at baseline and after propensity score matching

Characteristics	Before Propensity Score matching				After Propensity Score matching			
	Laparoscopic^1^ (*N* = 37,340)	Robotic-assisted^1^ (*N* = 17,369)	*p*-value^2^	SMD	Laparoscopic^1^ (*N* = 16,265)	Robotic-assisted^1^ (*N* = 16,265)	*p*-value^2^	SMD
Age Groups			**0.006**	0.029			0.65	0.01
18–44	19,407 (52.0%)	8,801 (50.7%)			8,212 (50.5%)	8,269 (50.8%)		
45–64	16,183 (43.3%)	7,684 (44.2%)			7,212 (44.3%)	7,187 (44.2%)		
≥ 65	1,750 (4.7%)	884 (5.1%)			841 (5.2%)	809 (5.0%)		
Gender			0.17	0.017			0.699	0.009
Female	31,978 (85.6%)	14,780 (85.1%)			13,908 (85.5%)	13,854 (85.2%)		
Male	5,357 (14.3%)	2,588 (14.9%)			2,356 (14.5%)	2,410 (14.8%)		
Unknown	5 (0.0%)	1 (0.0%)			1 (0.0%)	1 (0.0%)		
Race			**< 0.001**	0.128			0.187	0.024
White	24,094 (64.5%)	11,320 (65.2%)			10,542 (64.8%)	10,599 (65.2%)		
Black	7,784 (20.8%)	3,317 (19.1%)			3,302 (20.3%)	3,163 (19.4%)		
Other	4,228 (11.3%)	1,735 (10.0%)			1,625 (10.0%)	1,662 (10.2%)		
Unknown	1,234 (3.3%)	997 (5.7%)			796 (4.9%)	841 (5.2%)		
Hispanic Ethnicity			**< 0.001**	0.143			0.189	0.02
Yes	4,796 (12.8%)	2,893 (16.7%)			2,547 (15.7%)	2,631 (16.2%)		
No	28,293 (75.8%)	13,052 (75.1%)			12,416 (76.3%)	12,276 (75.5%)		
Unknown	4,251 (11.4%)	1,424 (8.2%)			1,302 (8.0%)	1,358 (8.3%)		
Marital status			**< 0.001**	0.089			0.225	0.023
Married	18,096 (48.5%)	8,741 (50.3%)			8,151 (50.1%)	8,184 (50.3%)		
Other	2,415 (6.5%)	779 (4.5%)			674 (4.1%)	744 (4.6%)		
Single	16,355 (43.8%)	7,620 (43.9%)			7,219 (44.4%)	7,126 (43.8%)		
Unknown	474 (1.3%)	229 (1.3%)			221 (1.4%)	211 (1.3%)		
Insurance Type			**< 0.001**	0.046			0.961	0.006
Commercial/Private	20,941 (56.1%)	9,420 (54.2%)			8,887 (54.6%)	8,849 (54.4%)		
Medicaid	9,310 (24.9%)	4,353 (25.1%)			4,063 (25.0%)	4,090 (25.1%)		
Medicare	4,688 (12.6%)	2,401 (13.8%)			2,231 (13.7%)	2,226 (13.7%)		
Others	2,401 (6.4%)	1,195 (6.9%)			1,084 (6.7%)	1,100 (6.8%)		
Charlson Comorbidity Score			**< 0.001**	0.037			0.503	0.013
0	15,790 (42.3%)	7,659 (44.1%)			7,219 (44.4%)	7,143 (43.9%)		
1	13,060 (35.0%)	5,882 (33.9%)			5,523 (34.0%)	5,516 (33.9%)		
≥ 2	8,490 (22.7%)	3,828 (22.0%)			3,523 (21.7%)	3,606 (22.2%)		
BMI type^3^			**< 0.001**	0.054			0.761	0.012
30–39	8,145 (21.8%)	4,164 (24.0%)			3,833 (23.6%)	3,854 (23.7%)		
40–49	19,838 (53.1%)	8,854 (51.0%)			8,431 (51.8%)	8,342 (51.3%)		
50–59	7,434 (19.9%)	3,465 (19.9%)			3,193 (19.6%)	3,238 (19.9%)		
60 and above	1,923 (5.1%)	886 (5.1%)			808 (5.0%)	831 (5.1%)		
Obesity related comorbidities								
Diabetes	11,663 (31.2%)	5,257 (30.3%)	**0.023**	0.021	4,964 (30.5%)	4,938 (30.4%)	0.763	0.003
Gastroesophageal reflux disease	23,617 (63.2%)	11,261 (64.8%)	**< 0.001**	0.033	10,555 (64.9%)	10,523 (64.7%)	0.719	0.004
Hypertension	20,892 (56.0%)	9,525 (54.8%)	**0.015**	0.022	9,018 (55.4%)	8,945 (55.0%)	0.422	0.009
Obstructive sleep Apnea	17,673 (47.3%)	8,467 (48.7%)	**0.002**	0.028	7,825 (48.1%)	7,893 (48.5%)	0.457	0.008
Hyperlipidemia	9,597 (25.7%)	4,335 (25.0%)	0.065	0.017	4,141 (25.5%)	4,082 (25.1%)	0.459	0.008
Liver disease	7,395 (19.8%)	2,923 (16.8%)	**< 0.001**	0.077	2,655 (16.3%)	2,788 (17.1%)	0.05	0.022
Hiatal hernia	195 (0.5%)	77 (0.4%)	**0.248**	0.011	66 (0.4%)	72 (0.4%)	0.67	0.006
MS DRG^3^			**< 0.001**	0.079			0.623	0.011
620 (O.R. procedures for obesity with CC)	5,737 (15.4%)	2,822 (16.2%)			2,569 (15.8%)	2,630 (16.2%)		
621 (O.R. procedures for obesity without CC/MCC)	29,636 (79.4%)	13,334 (76.8%)			12,620 (77.6%)	12,550 (77.2%)		
Other	1,967 (5.3%)	1,213 (7.0%)			1,076 (6.6%)	1,085 (6.7%)		
Hernia repair concomitant			**< 0.001**	0.065			0.843	0.01
Hiatal hernia repair	7,264 (19.5%)	3,819 (22.0%)			3578 (22.0%)	3532 (21.7%)		
Ventral hernia	938 (2.5%)	388 (2.2%)			357 (2.2%)	361 (2.2%)		
Hiatal hernia repair and ventral hernia repair	279 (0.7%)	107 (0.6%)			106 (0.7%)	97 (0.6%)		
No hernia repair	28,859 (77.3%)	13,055 (75.2%)			12,224 (75.2%)	12,275 (75.5%)		
Cholecystectomy concomitant	947 (2.5%)	505 (2.9%)	**0.013**	0.023	488 (3.0%)	463 (2.8%)	0.43	0.009
Lysis of adhesions	3,836 (10.3%)	1,958 (11.3%)	**< 0.001**	0.032	1,851 (11.4%)	1,820 (11.2%)	0.599	0.006
Nicotine use	10,084 (27.0%)	4,625 (26.6%)	0.359	0.009	4,282 (26.3%)	4,343 (26.7%)	0.451	0.008
Stapler use during surgery			**< 0.001**	0.339			0.149	0.022
No stapler	8,606 (23.0%)	4,816 (27.7%)			4,348 (26.7%)	4,477 (27.5%)		
Stapler without reinforcement	22,127 (59.3%)	11,370 (65.5%)			10,776 (66.3%)	10,609 (65.2%)		
Stapler with reinforcement	6,607 (17.7%)	1,183 (6.8%)			1,141 (7.0%)	1,179 (7.2%)		
Surgeon specialty			0.07	0.021			0.729	0.009
General surgery	33,128 (88.7%)	15,327 (88.2%)			14,350 (88.2%)	14,341 (88.2%)		
Other	3,910 (10.5%)	1,918 (11.0%)			1,806 (11.1%)	1,803 (11.1%)		
Unknown	302 (0.8%)	124 (0.7%)			109 (0.7%)	121 (0.7%)		
Surgeon volumes^4^			**< 0.001**	0.449			**0.022**	0.031
Low Volume (≤ 13)	4,879 (13.1%)	4,359 (25.1%)			3,657 (22.5%)	3,768 (23.2%)		
Medium Volume (14–51)	13,107 (35.1%)	7,534 (43.4%)			7,336 (45.1%)	7,089 (43.6%)		
High Volume (52–300)	19,354 (51.8%)	5,476 (31.5%)			5,272 (32.4%)	5,408 (33.2%)		
Hospital teaching status			**< 0.001**	0.139			0.28	0.012
Teaching	19,288 (51.7%)	10,170 (58.6%)			9,577 (58.9%)	9,480 (58.3%)		
Non-Teaching	18,052 (48.3%)	7,199 (41.4%)			6,688 (41.1%)	6,785 (41.7%)		
Region			**< 0.001**	0.092			0.911	0.001
Urban	35,645 (95.5%)	16,213 (93.3%)			15,186 (93.4%)	15,192 (93.4%)		
Rural	1,695 (4.5%)	1,156 (6.7%)			1,079 (6.6%)	1,073 (6.6%)		
Geographic Region			**< 0.001**	0.154			0.452	0.018
Midwest	9,843 (26.4%)	4,117 (23.7%)			3,846 (23.6%)	3,932 (24.2%)		
Northeast	7,455 (20.0%)	3,578 (20.6%)			3,260 (20.0%)	3,292 (20.2%)		
South	14,143 (37.9%)	7,624 (43.9%)			7,222 (44.4%)	7,081 (43.5%)		
West	5,899 (15.8%)	2,050 (11.8%)			1,937 (11.9%)	1,960 (12.1%)		
Hospital Bed Size			**< 0.001**	0.199			0.228	0.023
0-199	8,156 (21.8%)	2,790 (16.1%)			2,568 (15.8%)	2,638 (16.2%)		
200–299	6,525 (17.5%)	4,150 (23.9%)			3,871 (23.8%)	3,767 (23.2%)		
300–499	9,885 (26.5%)	4,823 (27.8%)			4,405 (27.1%)	4,517 (27.8%)		
500+	12,774 (34.2%)	5,606 (32.3%)			5,421 (33.3%)	5,343 (32.8%)		
Hospital Volumes^5^			**< 0.001**	0.394			0.335	0.016
Low Volume (≤ 72)	8,453 (22.6%)	5,925 (34.1%)			5,368 (33.0%)	5,337 (32.8%)		
Medium Volume (73–171)	12,236 (32.8%)	6,827 (39.3%)			6,465 (39.7%)	6,379 (39.2%)		
High Volume (172–510)	16,651 (44.6%)	4,617 (26.6%)			4,432 (27.2%)	4,549 (28.0%)		
Year of Admission			**< 0.001**	0.392			**0.001**	0.045
2020	8,843 (23.7%)	2,131 (12.3%)			2,359 (14.5%)	2,115 (13.0%)		
2021	9,889 (26.5%)	3,746 (21.6%)			3,528 (21.7%)	3,648 (22.4%)		
2022	10,474 (28.1%)	5,389 (31.0%)			5,053 (31.1%)	5,064 (31.1%)		
2023	8,134 (21.8%)	6,103 (35.1%)			5,325 (32.7%)	5,438 (33.4%)		

1. n (%)

2. Chi-square test (highlighted *p* < 0.05)

3. BMI, Body Mass Index; MS-DRG, Medicare Severity Diagnosis Related Groups; CC, Complication and Comorbidity; MCC, Major Complication and Comorbidity; O.R., Operating Room

4. Surgeon volumes; Median number of procedures for low volume: 6, median number of procedures for medium volume: 30, median number of procedures for high volume: 89

5. Hospital volumes; Median number of procedures for low volume: 49, median number of procedures for medium volume: 114, median number of procedures for high volume: 264

After applying PSM, covariates were balanced, with SMD < 0.1 (Appendix G) indicating a negligible difference. Residual imbalance in surgeon volume and admission year was adjusted further with regression model. PSM resulted in 16,265 in each of the LAP-RYGB and RAS-RYGB cohorts (Table 1). The characteristics after PSM matching are noted in Table 1.

For the outcomes in Table 2, RAS-RYGB was associated with lower odds of conversion to open surgery (OR, 0.48; 95% CI, 0.32–0.73) and ICU admission (OR, 0.57; 95% CI, 0.45–0.73) compared with LAP-RYGB. RAS-RYGB was also associated with a shorter length of stay, reflected by lower odds of being in a higher length-of-stay category (OR, 0.85; 95% CI, 0.81–0.89), and fewer 30-day reencounters (OR, 0.87; 95% CI, 0.82–0.91). Blood transfusions were less frequent among RAS-RYGB patients (OR, 0.77; 95% CI, 0.63–0.95). Operating room time was longer for RAS-RYGB, with a mean difference of 17.5 min (95% CI, 16.1–18.9). Bowel obstruction was more frequent following RAS-RYGB (OR, 1.25; 95% CI, 1.01–1.54). Other complications, such as anastomotic leak, bleeding, sepsis, and surgical site infection were similar between groups during index and 30 days post-surgery.

**Table 2:** Perioperative Outcomes in the Propensity Score Matched Cohort

Outcomes	Laparoscopic RYGB (*N* = 16,265)	Robotic assisted RYGB(*N* = 16,265)	Effect Estimates (95% CI)^1^	*p*-value
Conversion, n (%)	69 (0.4%)	33 (0.2%)	**0.48 (0.32**,** 0.73)**	**0.001**
ICU admissions, n (%)	175 (1.1%)	100 (0.6%)	**0.57 (0.45**,** 0.73)**	**< 0.001**
Operating room time, mins				
Mean (SD)	177.02 (62.01)	194.40 (72.44)	**17.50 (16.07**,** 18.93)**	**< 0.001**
Median (Q1, Q3)	170 (135, 210)	180 (150, 240)		
Length of stay, n (%)			**0.85 (0.81**,** 0.89)**	**< 0.001**
1 day	9,724 (59.8%)	10,374 (63.8%)		
2–4 days	6,210 (38.2%)	5,613 (34.5%)		
> 4 days	331 (2.0%)	278 (1.7%)		
Reencounters 30 days, n (%)	4,197 (25.8%)	3,761 (23.1%)	**0.87 (0.82**,** 0.91)**	**< 0.001**
Readmissions 30 days, n (%)	645 (4.0%)	679 (4.2%)	1.06 (0.95, 1.18)	0.318
Reoperations 30 days, n (%)	282 (1.7%)	264 (1.6%)	0.94 (0.79, 1.11)	0.457
Complications at index and 30d post, n (%)				
Anastomotic leak	218 (1.3%)	207 (1.3%)	0.95 (0.79, 1.15)	0.606
Bleeding	486 (3.0%)	486 (3.0%)	1.00 (0.88, 1.14)	0.996
Blood transfusions	205 (1.3%)	159 (1.0%)	**0.77 (0.63**,** 0.95)**	**0.014**
Bowel obstruction	161 (1.0%)	200 (1.2%)	**1.25 (1.01**,** 1.54)**	**0.037**
Surgical Site Infection	134 (0.8%)	143 (0.9%)	1.07 (0.84, 1.36)	0.572
Sepsis	115 (0.7%)	121 (0.7%)	1.06 (0.82, 1.37)	0.664

^1^Effect estimates are odds ratios for binary outcomes and mean differences for continuous outcomes, with 95% CIs. Odds ratios compare Robotic-assisted RYGB with Laparoscopic-RYGB as the reference group. Mean differences represent Robotic-assisted RYGB minus Laparoscopic RYGB

Subgroup matched comparison (Appendix H) excluding patients with concomitant hernia repairs (*n* = 12,216) had results consistent with the main analysis. Compared with LAP-RYGB, RAS-RYGB was associated with lower conversions (OR, 0.63; 95% CI, 0.42–0.93), shorter length of stay (OR, 0.88; 95% CI, 0.84–0.93), fewer 30-day reencounters (OR, 0.91; 95% CI, 0.86–0.97), and lower blood transfusion rates (OR, 0.72; 95% CI, 0.56–0.91). However, bowel obstruction was more frequent among RAS-RYGB patients (1.0% vs. 0.8%; OR, 1.32; 95% CI, 1.02–1.72). In patients with class 3 obesity (*n* = 12,419), the analysis also indicated consistent results with the main analysis (Appendix I).

## Discussion

Using national population-based data, this study assessed trends in the utilization of LAP and RAS approaches for RYGB surgery in recent years. The utilization of RAS increased from 2020 to 2023, while the rate of LAP correspondingly decreased. These findings emphasize the increasing uptake of RAS-RYGB, which is concordant with previous studies [22, 23]. This shift may reflect broader acceptance and integration of RAS technology in MBS [10, 24]. Later era of RAS have reported different outcomes due to newer platforms and refined stapling technology associated with reduced pulmonary complications, readmissions, reoperations, and shorter length of stay compared with earlier RAS periods [23]. Additionally, recently established global benchmarks suggest RAS MBS may enhance surgical safety compared with LAP approaches [25]. 

Our propensity score-matched analysis identified several differences between the outcomes of RAS-RYGB and LAP-RYGB. RAS-RYGB was associated with a modest reduction in LOS compared with LAP-RYGB, reflected by lower odds of being in a higher length-of-stay category and a modestly greater proportion of patients discharged after a 1-day hospitalization. In our analysis RAS-RYGB was associated with fewer ICU admissions, blood transfusions and conversion to open for RAS as compared to LAP. The shorter length of stay is consistent with the findings from a single center study that identified shorter mean hospital stay for RAS-RYGB compared to LAP-RYGB [26]. This length of stay difference is further supported by a meta-analysis that reported RAS-RYGB is associated with fewer anastomotic stricture events, reoperations, and a decreased length of hospital stay compared with LAP-RYGB [13]. In other studies, RAS-RYGB was associated with equivalent LOS compared to LAP-RYGB [27, 28]. Despite similar rates of anastomotic leaks, bleeding, surgical site infections and sepsis, we observed lower ICU admissions for RAS-RYGB. While our matching strategy adjusted for major hospital-level characteristics such as bed size and teaching versus non-teaching status, the observed differences in ICU admission rates may also reflect institutional practice patterns that extend beyond these measured variables. Hospitals differ in their care-delivery models, including nurse-to-patient ratios and dedicated ICU physicians and staff, which can influence whether a patient is admitted to the ICU, independent of clinical severity or surgical approach.

Our finding of lower perioperative blood transfusions for RAS‑RYGB is consistent with published evidence. A single-center study by Beckmann et al. reported fewer 30-day complications classified as Clavien-Dindo II-V for RAS-RYGB compared to LAP-RYGB [29]. A Metabolic and Bariatric Surgery Accreditation Quality Improvement Project (MBSAQIP) study reported lower perioperative blood transfusions and similar unplanned ICU admissions for RAS-RYGB as compared to LAP-RYGB [30]. Furthermore, in our study, the conversion rate was lower for RAS-RYGB (0.2%) compared to LAP-RYGB (0.4%). While this trend suggests an advantage of the RAS approach, it differs from findings in the literature which reported similar conversion rates between the two techniques [26, 30]. These differences in outcomes may reflect evolving surgical practices or operative techniques, as our analysis includes more recent clinical practice patterns from 2020 to 2023.

Bowel obstruction was more frequently observed following RAS-RYGB. Although the reasons for this finding are unclear, bowel obstruction after RYGB has been associated with internal hernias, adhesions, and other anatomic strictures of the bypass reconstruction [31]. RAS-RYGB patients had a higher mean operative time (194.4) compared to LAP-RYGB (177.02 min). However, reporting methods of operative time can vary by hospital. This finding is corroborated by studies that have reported longer OR time for RAS than LAP-RYGB procedures [28, 32]. In contrast, a single surgeon, single center study RAS-RYGB had shorter operating time than LAP-RYGB [27]. Evidence suggests that the operation time can reduce significantly as surgeons progress along the learning curve [33, 34]. 

Several outcomes differed statistically between the two approaches. However, given the large sample size, statistical significance does not necessarily reflect clinical relevance. For a number of outcomes, including length of stay, the absolute differences were small and may have limited bearing on individual patient care or surgical decision-making. Conversely, outcomes such as conversion to open surgery, ICU admission, and blood transfusion, bowel obstructions though also modest in absolute terms, are clinically meaningful events that may carry greater weight for patients and clinicians. Singular, high impact events can affect payor and societal quality program accreditation leading to potential changes in patient access. Therefore, these findings should be interpreted according to the absolute magnitude, odds ratio and clinical importance of each outcome rather than statistical significance alone.

## Limitations

Given the inherent limitations associated with the retrospective design of our study, it is important to interpret the results with appropriate consideration. Firstly, there is a risk of misclassification bias of RAS-RYGB to LAP-RYGB cases due to under coding of RAS. However, we further utilize billing description terms to help identify RAS from LAP and open surgery beyond the RAS CPT/ICD codes, which may mitigate the impact of misclassification. Although the dataset covers approximately 25% of inpatient hospital discharges nationally, this could limit generalizability of results due to hospital selection bias. However, the distributions of hospitals such as rural-urban ratio and teaching status in PHD are comparable with American Health Association Database (AHAD) which includes all US hospitals [15, 33]. 

While propensity score matching with patient, hospital and provider characteristics adjusts for confounding variables that are available, there is a possibility of unmeasured confounding bias. PHD is a hospital administrative database. Therefore, comorbidities, procedures, and complications depend on ICD/CPT codes and hospital documentation practices. Granular clinical details related to operative complexity, perioperative decision-making, and surgeon experience were not available. We used each surgeon’s RYGB volume in the prior year as a proxy for experience, although this measure was limited to procedures performed within the same hospital and may underestimate experience for surgeons practicing across multiple sites. The reporting of OR time varies by hospital. In PHD it is reported in incremental blocks and may reflect wheels in and out time, or scheduled time, or patient room time. In addition, this study focused on perioperative clinical outcomes and did not include direct cost data or longer-term outcomes such as weight loss or comorbidity resolution. Despite these limitations, our study provides valuable insights into comparative outcomes of LAP and RAS in a large diverse population. Notably, it reflects current clinical practice, offering a timely perspective on evolving surgical trends and outcomes.

## Conclusion

In conclusion, RAS-RYGB utilization increased from 2020 to 2023 in this contemporary all-payer hospital discharge database. RAS-RYGB was associated with lower odds of conversion to open surgery, ICU encounters, blood transfusion and modestly shorter LOS, but also longer operative times and higher bowel obstruction rates. These findings contribute to the growing body of evidence helping to explain provider adoption of RAS techniques in MBS. Future research should assess long-term clinical outcomes when comparing RAS-RYGB with LAP-RYGB .

## Supplementary Information

Below is the link to the electronic supplementary material.

Supplementary Material 1

## Data Availability

The data that support the findings of this study are available from Premier Healthcare Database (https://offers.pinc-ai.com/Premier-Healthcare-Database-Download.html) but restrictions apply to the availability of these data, which were used under license for the current study, and so are not publicly available. Data is, however, available from the authors upon reasonable request and with permission of Premier Inc.
