# Supplementary material for: Comparative analysis of robotic assisted versus laparoscopic Roux-en-Y gastric bypass: a retrospective cohort study
Source: J Robot Surg. 2026 Jul 30;20(1):771. doi: 10.1007/s11701-026-03734-6 (PMC13424403; doi:10.1007/s11701-026-03734-6)
Supplement: Supplementary file 1 — Supplementary Material 1 [file 11701_2026_3734_MOESM1_ESM.docx]

Appendix A: Codes for gastric bypass procedures by modality

| **Modality** | **CPT/ HCPCS** | **ICD-10 Codes** |
| --- | --- | --- |
| Open | 43846, 43847 | 0D16079, 0D1607A, 0D1607B, 0D1607L, 0D160J9, 0D160JA, 0D160JB, 0D160JL, 0D160K9, 0D160KA, 0D160KB, 0D160KL, 0D160Z9, 0D160ZA, 0D160ZB, 0D160ZL0, D16879, 0D1687A, 0D1687B, 0D1687L, 0D168J9, 0D168JA, 0D168JB, 0D168JL, 0D168K9, 0D168KA, 0D168KB, 0D168KL, 0D168Z9, 0D168ZA, 0D168ZB, 0D168ZL |
| Laparoscopy | 43644, 43645 | 0D16479, 0D1647A, 0D1647B, 0D1647L, 0D164J9, 0D164JA, 0D164JB, 0D164JL, 0D164K9, 0D164KA, 0D164KB, 0D164KL, 0D164Z9, 0D164ZA, 0D164ZB, 0D164ZL |
| Robotic | S2900 | 8E0**CZ, Keywords in billing: “Da Vinci”, “Endowrist”, ”Robotic”, ”Rob” |
| Conversions to Open |  | Z53.31, Z53.39 |

Appendix B: ICD and CPT codes for history of bariatric surgery

| **Exclusion criteria** | **CPT** | **ICD-10 Codes** |
| --- | --- | --- |
| Revision of Bariatric Surgery | 43771, 43772, 43773, 43774, 43848, 43860, 43865, 43886, 43887, 43888 | ICD-10-PCS: 0DP60CZ, 0DP63CZ, 0DP64CZ, 0DP67CZ, 0DP68CZ, 0DW60CZ, 0DW63CZ, 0DW64CZ, 0DW67CZ, 0DW68CZ |
| History of bariatric surgery | -- | ICD-10-CM: K95.01, K95.09, K95.81, K95.89 |
| Diagnosis of malignancy of digestive track | -- | ICD-10-CM: C15, C16, C17, C18, C19, C20, C21, C22, C23, C24, C25, C26, C49, C7A, C7B, C78, C48, D01 |

Appendix C: Variables used for propensity score matching.

| **Characteristics** | **Variable Description** | **Categories** |
| --- | --- | --- |
| Patient comorbidities | BMI/obesity | BMI between 30 and 39  BMI between 40 and 49  BMI between 50 and 59  BMI between 60 and above |
|  | Diabetes | 1, 0 |
|  | Liver disease | 1, 0 |
|  | GERD | 1,0 |
|  | Hypertension | 1,0 |
|  | Hyperlipidemia | 1,0 |
|  | Sleep Apnea | 1,0 |
|  | Hiatal hernia diagnosis | 1,0 |
|  | Use of nicotine | 1,0 |
| Concomitant procedures | Hernia repair | Hiatal hernia repair, Ventral hernia, Hiatal hernia repair and ventral hernia repair, No hernia repair |
|  | Cholecystectomy | 1,0 |
|  | Lysis of adhesions | 1, 0 |
| Type of surgical stapler | Stapler use during surgery | Stapler with reinforcement, stapler without reinforcement, no use of a stapler |
| Patient characteristics | Age | At index: 18-54, 55-64, >=65 |
|  | Gender | Female, male, unknown |
|  | Marital status | Single, Married, Other, Unknown |
|  | Race | Black, White, Other, Unknown |
|  | Hispanic ethnicity | Yes, No |
|  | Charlson Comorbidity Index | 0, 1, ≥2 |
|  | Admission year | 2020, 2021, 2022, 2023 |
|  | MS-DRG | 620, 621, Other |
| Hospital characteristics | Payor | Primary payer (Medicare, Medicaid, Commercial, Others) |
|  | Hospital census region | West, Northeast, Midwest, South |
|  | Hospital volume | Low Volume,  Medium Volume,  High Volume, |
|  | Hospital number of beds | 0-199, 200-299, 300-499, 500+ |
|  | Hospital teaching status | Teaching, Non-Teaching |
|  | Hospital urban rural status | Urban, Rural |
| Surgeon characteristics | Surgeon volume | Low volume,  Medium volume,  High Volume |
|  | Surgeon specialty | General (GS), Other, Unknown |

Appendix D: Procedure and diagnosis codes for comorbidities

| **Comorbidities** | **ICD 10/CPT codes** |
| --- | --- |
| BMI/obesity >=30 | Z68.3, Z68.30, Z68.31, Z68.32, Z68.33, Z68.34, Z68.35, Z68.36, Z68.37, Z68.38, Z68.39, Z68.41, Z68.42, Z68.43, Z68.44, Z68.45 |
| Lysis of adhesions procedure | CPT: 44005, 44180  ICD: 0DN, 0FN, 0TN, 0UN |
| Lysis of adhesions diagnosis | K56.5, K66.0, N73.6, N99.4, Q43.3 |
| Diabetes | E10, E11, E13 |
| Liver disease | B18, K70, K71, K72, K73, K74, K76, Z94, I85, I86 |
| GERD | K21, K21.0, K21.00, K21.01, K21.9 |
| Hypertension | I10, I11, I11.0, I11.9, I12, I12.0, I12.9, I13, I13.0, I13.10, I13.11, I13.2, I15, I15.0, I15.1, I15.2, I15.8, I15.9, I16, I16.0, I16.1, I16.9 |
| Hiatal hernia repair procedure | CPT: 43280, 43281, 43282, 43325, 43327, 43328, 43332, 43333, 43334, 43335, 43336, 43337  ICD: 0BQR0ZZ, 0BQR3ZZ, 0BQR4ZZ, 0BQS0ZZ, 0BQS3ZZ, 0BQS4ZZ, 0BUR07Z, 0BUR0JZ, 0BUR0KZ, 0BUR47Z, 0BUR4JZ, 0BUR4KZ, 0BUS07Z, 0BUS0JZ, 0BUS0KZ, 0BUS47Z, 0BUS4JZ, 0BUS4KZ, 0BQT4ZZ, 0BRT47Z, 0BRT4JZ, 0BRT4KZ, 0BUT47Z, 0BUT4JZ, 0BUT4KZ, 0BQT0ZZ, 0BQT3ZZ, 0BRT07Z, 0BRT0JZ, 0BRT0KZ, 0BUT07Z, 0BUT0JZ, 0BUT0KZ, 0BQRS4ZZ, 0BQRS0ZZ |
| Ventral hernia repair procedure | CPT: 49560, 49561, 49585, 49587, 49652, 49653, 49654, 49655, 49570, 49572 ICD: 0WQF0ZZ, 0WQF3ZZ, 0WQF4ZZ, 0WQFXZZ, 0WUF07Z, 0WUF0JZ, 0WUF0KZ, 0WUF47Z, 0WUF4JZ, 0WUF4KZ |
| Cholecystectomy | CPT: 47562, 47563, 47564, 47600, 47605, 47610, 47612, 47620  ICD: 0FB44ZZ, 0FT44ZZ, 0FB40ZZ, 0FT40ZZ |
| Sleep Apnea | G47.3, G47.30, G47.31, G47.32, G47.33, G47.34, G47.35, G47.36, G47.37, G47.39 |
| Hyperlipidemia | E78, E78.41, E78.49, E78.5 |
| Nicotine use | F17.200, F17.201, F17.203, F17.208, F17.209, F17.210, F17.211, F17.213, F17.218, F17.219, F17.220, F17.221, F17.223, F17.228, F17.229, F17.290, F17.291, F17.293, F17.298, F17.299, O99.330, O99.331, O99.332, O99.333, O99.334, O99.335, T65.211A, T65.212A, T65.213A, T65.214A, T65.221A, T65.222A, T65.223A, T65.224A, T65.291A, T65.292A, T65.293A, T65.294A, T65.211D, T65.212D, T65.213D, T65.214D, T65.221D, T65.222D, T65.223D, T65.224D, T65.291D, T65.292D, T65.293D, T65.294D, T65.211S, T65.212S, T65.213S, T65.214S, T65.221S, T65.222S, T65.223S, T65.224S, T65.291S, T65.292S, T65.293S, T65.294S, Z72.0, Z87.891 |

Appendix E: Procedure and diagnosis codes for complications

| **Variable Description** | **ICD/CPT codes** |
| --- | --- |
| Bleeding | K91.61, K91.62, K91.840, K91.841, K91.870, K91.871, D62, K92.2, R58, T79.2XXA |
| Surgical site infection | 0H98X, 0H9AX, 0H9BX, 0H9CX, 0H9DX, 0H9EX, 0H9FX, 0H9GX, 0H9HX, 0H9JX, 0H9KX, 0H9LX, 0H9MX, 0H9NX, 0HB, 0J9, 0JB, 0W9, 0X9, 0Y9 |
| Bowel obstruction | K56.5, K56.50, K56.51, K56.52, K56.60, K56.600, K56.601, K56.609, K56.69, K56.690, K56.691, K56.699 |
| Anastomotic leak | K31.6, K63.0, K63.2, K65.0, K65.1, K68.11, N32.1, N82.4, T81.4XXA, T81.83XA, Y83.2 |
| Blood transfusion | CPT: 36430  ICD: 30233H0, 30233H1, 30233K1, 30233L1, 30233M1, 30233N0, 30233N1, 30233P1, 30233R1, 30243H0, 30243H1, 30243J1, 30243K1, 30243L1, 30243M1, 30243N0, 30243N1, 30243P1,  30243R1, 30253H0, 30253H1, 30253K1, 30253L1, 30253M1, 30253N0, 30253N1, 30253P1, 30253Q1, 30253R1, 30253T1, 30263H0, 30263H1, 30263K0, 30263K1, 30263L0, 30263L1, 30263M1, 30263N0, 30263N1, 30263P0, 30263P1, 30263R0, 30263R1 |
| Sepsis | A40, A41, A42, R65, R78, T81 |

Appendix F: STROBE Statement—Checklist of items that should be included in reports of ***cohort studies***

|  | **Item No** | **Recommendation** | **Page No** |
| --- | --- | --- | --- |
| **Title and abstract** | 1 | (*a*) Indicate the study’s design with a commonly used term in the title or the abstract | Title and Abstract |
|  |  | (*b*) Provide in the abstract an informative and balanced summary of what was done and what was found | Abstract |
| **Introduction** | | |  |
| Background/rationale | 2 | Explain the scientific background and rationale for the investigation being reported | 1 |
| Objectives | 3 | State specific objectives, including any prespecified hypotheses | 1 |
| **Methods** | | |  |
| Study design | 4 | Present key elements of study design early in the paper | 2 |
| Setting | 5 | Describe the setting, locations, and relevant dates, including periods of recruitment, exposure, follow-up, and data collection | 2-3 |
| Participants | 6 | (*a*) Give the eligibility criteria, and the sources and methods of selection of participants. Describe methods of follow-up | 2-3 |
|  |  | (*b*) For matched studies, give matching criteria and number of exposed and unexposed | 4-6 |
| Variables | 7 | Clearly define all outcomes, exposures, predictors, potential confounders, and effect modifiers. Give diagnostic criteria, if applicable | 4-6 |
| Data sources/ measurement | 8* | For each variable of interest, give sources of data and details of methods of assessment (measurement). Describe comparability of assessment methods if there is more than one group | 4-6 |
| Bias | 9 | Describe any efforts to address potential sources of bias | 9-10 |
| Study size | 10 | Explain how the study size was arrived at | Fig 1 |
| Quantitative variables | 11 | Explain how quantitative variables were handled in the analyses. If applicable, describe which groupings were chosen and why | 4-6 |
| Statistical methods | 12 | (*a*) Describe all statistical methods, including those used to control for confounding | 4-5 |
|  |  | (*b*) Describe any methods used to examine subgroups and interactions | 4-5 |
|  |  | (*c*) Explain how missing data were addressed | 4-5 |
|  |  | (*d*) If applicable, explain how loss to follow-up was addressed | 4-5 |
|  |  | (*e*) Describe any sensitivity analyses | -- |
| **Results** | | |  |
| Participants | 13* | (a) Report numbers of individuals at each stage of study—eg numbers potentially eligible, examined for eligibility, confirmed eligible, included in the study, completing follow-up, and analysed | 6 |
|  |  | (b) Give reasons for non-participation at each stage | 6 |
|  |  | (c) Consider use of a flow diagram | Fig 1. |
| Descriptive data | 14* | (a) Give characteristics of study participants (eg demographic, clinical, social) and information on exposures and potential confounders | Table 1 |
|  |  | (b) Indicate number of participants with missing data for each variable of interest | Table 1 |
|  |  | (c) Summarise follow-up time (eg, average and total amount) | 3,5,7 |
| Outcome data | 15* | Report numbers of outcome events or summary measures over time | Table 2 |
| Main results | 16 | (*a*) Give unadjusted estimates and, if applicable, confounder-adjusted estimates and their precision (eg, 95% confidence interval). Make clear which confounders were adjusted for and why they were included | Page 5, Table 1-2 |
|  |  | (*b*) Report category boundaries when continuous variables were categorized | Table 1-2 |
|  |  | (*c*) If relevant, consider translating estimates of relative risk into absolute risk for a meaningful time period | -- |
| Other analyses | 17 | Report other analyses done—eg analyses of subgroups and interactions, and sensitivity analyses | Appendix H, I |
| **Discussion** | | |  |
| Key results | 18 | Summarise key results with reference to study objectives | 5-7 |
| Limitations | 19 | Discuss limitations of the study, taking into account sources of potential bias or imprecision. Discuss both direction and magnitude of any potential bias | 9-10 |
| Interpretation | 20 | Give a cautious overall interpretation of results considering objectives, limitations, multiplicity of analyses, results from similar studies, and other relevant evidence | 10 |
| Generalisability | 21 | Discuss the generalisability (external validity) of the study results | 10 |
| **Other information** | | |  |
| Funding | 22 | Give the source of funding and the role of the funders for the present study and, if applicable, for the original study on which the present article is based | Title page |

*Give information separately for exposed and unexposed groups.

Appendix G: Covariate balance analysis before and after propensity score matching using standardized mean differences.


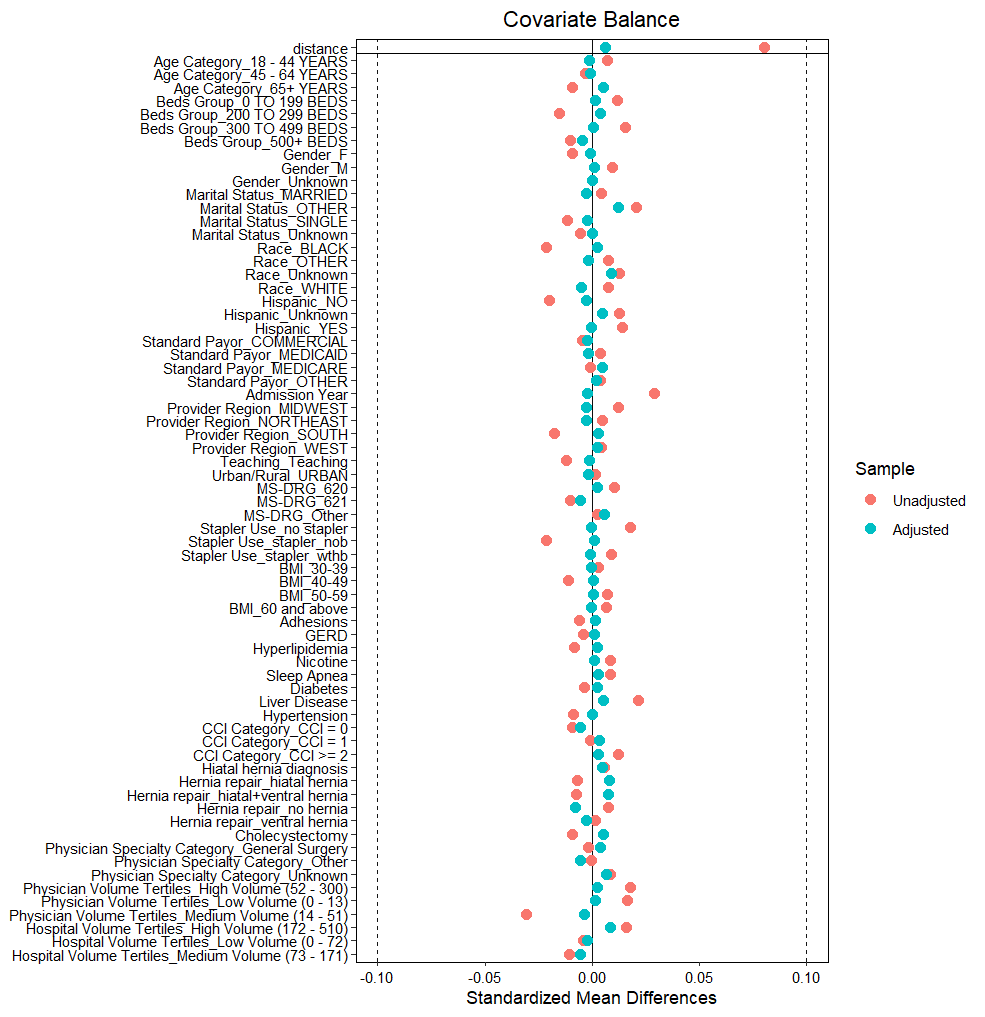


Appendix H: Bivariate analysis of propensity score matched outcomes in gastric bypass patients done without concomitant hernia repair

| **Outcomes** | **Laparoscopic (N=12,216)** | **Robotic-assisted**  **(N=12,216)** | **p-value^1^** |
| --- | --- | --- | --- |
| **Conversion, n (%)** | 66 (0.5%) | 41 (0.3%) | **0.02** |
| **ICU^2^ admissions, n (%)** | 141 (1.2%) | 84 (0.7%) | **<0.001** |
| **Operating room time, mins** |  |  |  |
| mean (SD) | 171.64 (59.35) | 189.75 (68.91) | **<0.001** |
| median (Q1, Q3) | 165 (132, 210) | 180 (150, 225) |  |
| **Length of Stay, days** |  |  |  |
| mean (SD) | 1.61 (1.52) | 1.56 (1.67) | **0.023** |
| median (Q1, Q3) | 1(1,2) | 1(1,2) |  |
| **Length of stay, n (%)** |  |  | **<0.001** |
| 1 day | 7,520 (61.6%) | 7,904 (64.7%) |  |
| 2-4 days | 4,463 (36.5%) | 4,122 (33.7%) |  |
| >4 days | 233 (1.9%) | 190 (1.6%) |  |
| **Reencounters 30 days, n (%)** | 2,958 (24.2%) | 2,738 (22.4%) | **0.001** |
| **Readmissions 30 days, n (%)** | 449 (3.7%) | 487 (4.0%) | 0.217 |
| **Reoperations 30 days, n (%)** | 189 (1.5%) | 174 (1.4%) | 0.459 |
| **Complications at index and 30d post, n (%)** |  |  |  |
| Anastomotic leak | 100 (0.8%) | 92 (0.8%) | 0.612 |
| Bleeding | 99 (0.8%) | 95 (0.8%) | 0.829 |
| Blood transfusions | 129 (1.1%) | 97 (0.8%) | **0.038** |
| Bowel obstruction | 95 (0.8%) | 117 (1.0%) | 0.147 |
| Surgical Site Infection | 69 (0.6%) | 68 (0.6%) | 1 |
| Sepsis | 45 (0.4%) | 48 (0.4%) | 0.835 |

*^1^Pearson chi-sq test for categorical variables and two sample t-test for continuous variables*

*^2^Intensive Care Unit*

Appendix I: Bivariate analysis of propensity score matched outcomes in gastric bypass patients among Class 3 BMI (>=40)

| **Outcomes** | **Laparoscopic RYGB (N=12,419)** | **Robotic-assisted RYGB (N=12,419)** | **p-value^1^** |
| --- | --- | --- | --- |
| Conversion, n (%) | 67 (0.5%) | 37 (0.3%) | **0.004** |
| ICU^2^admissions, n (%) | 131 (1.1%) | 81 (0.7%) | **0.001** |
| Operating room time, mins |  |  |  |
| mean (SD) | 176.13 (61.35) | 194.10 (72.00) | **<0.001** |
| median (Q1, Q3) | 169 (135, 210) | 180 (150, 240) |  |
| Length of Stay, days |  |  |  |
| mean (SD) | 1.62 (1.47) | 1.57 (1.56) | **0.014** |
| median (Q1, Q3) | 1 (1,2) | 1 (1,2) |  |
| Length of stay, n (%) |  |  | **<0.001** |
| 1 day | 7,502 (60.4%) | 7,951 (64.0%) |  |
| 2-4 days | 4,659 (37.5%) | 4,258 (34.3%) |  |
| >4 days | 258 (2.1%) | 210 (1.7%) |  |
| **Reencounters 30 days, n (%)** | 3119 (25.1%) | 2855 (23%) | **<0.001** |
| **Readmissions 30 days, n (%)** | 445 (3.6%) | 504 (4.1%) | 0.055 |
| **Reoperations 30 days, n (%)** | 200 (1.6%) | 199 (1.6%) | 1 |
| **Complications at index and 30d post, n (%)** |  |  |  |
| Anastomotic leak | 142 (1.1%) | 151 (1.2%) | 0.638 |
| Bleeding | 373 (3.0%) | 345 (2.8%) | 0.307 |
| Blood transfusions | 145 (1.2%) | 112 (0.9%) | **0.045** |
| Bowel obstruction | 102 (0.8%) | 133 (1.1%) | **0.049** |
| Surgical Site Infection | 99 (0.8%) | 108 (0.9%) | 0.577 |
| Sepsis | 81 (0.7%) | 92 (0.7%) | 0.445 |

*^1^Pearson chi-sq test for categorical variables and two sample t-test for continuous variables*

*^2^Intensive Care Unit*
